# Development and validation of a 1 K sika deer (*Cervus nippon*) SNP Chip

**DOI:** 10.1186/s12863-021-00994-z

**Published:** 2021-09-17

**Authors:** Huanhuan Fan, Tianjiao Wang, Yang Li, Huitao Liu, Yimeng Dong, Ranran Zhang, Hongliang Wang, Liyuan Shang, Xiumei Xing

**Affiliations:** 1 Key Laboratory of Molecular Biology of Special Economic Animals, Institute of Special Products, Chinese Academy of Agricultural Sciences, Changchun, 130112 China; 2 Jilin Animal Husbandry and Veterinary Research Institute Changchun, Changchun, 130112 China

**Keywords:** SNP chip, Sika deer, Red deer, Hybrid deer, Identification

## Abstract

**Background:**

China is the birthplace of the deer family and the country with the most abundant deer resources. However, at present, China’s deer industry faces the problem that pure sika deer and hybrid deer cannot be easily distinguished. Therefore, the development of a SNP identification chip is urgently required.

**Results:**

In this study, 250 sika deer, 206 red deer, 23 first-generation hybrid deer (F1), 20 s-generation hybrid deer (F2), and 20 third-generation hybrid deer (F3) were resequenced. Using the chromosome-level sika deer genome as the reference sequence, mutation detection was performed on all individuals, and a total of 130,306,923 SNP loci were generated. After quality control filtering was performed, the remaining 31,140,900 loci were confirmed. From molecular-level and morphological analyses, the sika deer reference population and the red deer reference population were established. The Fst values of all SNPs in the two reference populations were calculated. According to customized algorithms and strict screening principles, 1000 red deer-specific SNP sites were finally selected for chip design, and 63 hybrid individuals were determined to contain red deer-specific SNP loci. The results showed that the gene content of red deer gradually decreased in subsequent hybrid generations, and this decrease roughly conformed to the law of statistical genetics. Reaction probes were designed according to the screening sites. All candidate sites met the requirements of the Illumina chip scoring system. The average score was 0.99, and the MAF was in the range of 0.3277 to 0.3621. Furthermore, 266 deer (125 sika deer, 39 red deer, 56 F1, 29 F2,17 F3) were randomly selected for 1 K SNP chip verification. The results showed that among the 1000 SNP sites, 995 probes were synthesized, 4 of which could not be typed, while 973 loci were polymorphic. PCA, random forest and ADMIXTURE results showed that the 1 K sika deer SNP chip was able to clearly distinguish sika deer, red deer, and hybrid deer and that this 1 K SNP chip technology may provide technical support for the protection and utilization of pure sika deer species resources.

**Conclusion:**

We successfully developed a low-density identification chip that can quickly and accurately distinguish sika deer from their hybrid offspring, thereby providing technical support for the protection and utilization of pure sika deer germplasm resources.

**Supplementary Information:**

The online version contains supplementary material available at 10.1186/s12863-021-00994-z.

## Background

China has one of the largest and most diverse deer populations in the world. In a study on the genetic diversity of Chinese antler deer, Xing [1] proposed that there are 19 deer species in 10 genera, including sika deer, red deer, tufted deer, and white-lipped deer, in China. This diversity of deer resources is an important component of the special animal germplasm resources of China and represents an economically important resource. Among these deer, sika deer and red deer are two species belonging to the order *Artiodactyla*, family *Cervidae*, and genus *Cervus*. The high degree of homology between the genomes of these two deer species indicate that their degrees of reproductive isolation and genetic isolation are relatively small [2], and that they have not yet reached the stage of restricted or inhibited gene exchange [3]. In fact, fertile offspring can be produced in the wild and in captivity [4], and hybrid deer exhibit notable velvet quality traits and reproductive traits, indicating heterosis. To pursue greater economic benefits, cross-breeding was applied in the breeding process of antler deer, with the main hybridization method being crossing or progressive crossing between sika deer and red deer [5]. Specifically, the first generation of hybrids was crossed with sika deer to produce a second generation of hybrids, and the second generation of hybrids was crossed with sika deer to produce a third generation of hybrid deer. The phenotype of the second-generation hybrid deer was very similar to that of the sika deer, and the hybrids were difficult to distinguish with the naked eye, enabling the hybrid offspring and pure sika deer to intermingle. This intermingling has posed considerable challenges to the protection and utilization of pure sika deer. As a result, how to effectively identify and protect existing pure sika deer resources has become highly important.

Traditional identification of purebred sika deer is primarily based on morphological characteristics. Such characteristics are easily influenced by the environment and seasonal variation, the identification step is time-consuming, and the work is demanding. Thus, identification using phenotypic traits alone is not accurate, comprehensive or scientific. Subsequently, the identification of purebred sika deer evolved from relying on traditional phenotyping to employing DNA molecular marker technology. DNA is the basic carrier of biological genetic information. The DNA sequence in each organism is unique and can be used as a biological indicator. DNA molecular marker technology has extremely high application value [6], especially for some populations that are difficult to identify on the basis of their appearances, as molecular marker technology can be employed to identify them scientifically and accurately. According to the order of development, DNA molecular markers are divided into the first, second, and third generations. The first generation of DNA molecular markers is represented by restriction fragment length polymorphisms (RFLPs) and random amplified polymorphic DNA (RAPD), the second generation is represented by simple sequence repeats (SSRs), and the third generation is represented by expressed sequence tags (ESTs) and single nucleotide polymorphisms (SNPs) [7]. As a kind of DNA molecular marker, SNPs have the advantages of abundant polymorphisms, large quantities, stable genetics, fast detection, high quality, automatic labeling technology and large-scale detection. Moreover, the dimorphism of these markers is conducive to genotyping and is currently traceable. For these reasons, SNPs are currently the most important and effective genetic marker in use.

With the reduction in high-throughput sequencing costs and the development of SNP chips, whole-genome SNP chips have emerged. To date, several SNP chips have been developed in a variety of plants and animals, for example rice [8], grapes [9], the salmon [10], and in livestock species like the pig [11], the cattle [12], the horse [13], the goat [14], the sheep (Illumina Ovine 50 k SNP BeadChip [15] and Illumina Ovine High-Density (HD) SNP BeadChip [16]), the chicken [17], and also in other domestic species like the dog [18] and the cat [19]. SNP chips are important tools for genetic diversity analysis, variety relationship analysis, genome-wide association studies (GWASs), and quantitative trait identification [20]. In addition, SNP chips are also used for breed and species identification. For example, the SNP chip of *G. hirsutum* [21] contains 17,954 interspecific SNPs, which can accurately distinguish land cotton from sea island cotton. The chicken 55 K chip [22] can identify 13 native Chinese breeds of chickens. SNP chips are also widely used in population genomics research. For example, Canas et al., [20] used the Illumina Bovine 777 K HD Bead Chip to analyze the genetic diversity of 7 important breeds of native Spanish beef cattle. The resulting phylogenetic tree showed that the 7 breeds originated from two main groups, and the differences within the breeds were large. Dasilvl et al., [23] used a high-density SNP chip to detect mutations in 2175 robins and identified 41,029 copy number variations (CNVs). The characteristics of these CNVs reflected how robins evolve in constantly changing environments. Talenti [24] used the GoatSNP50 chip to sequence data from 109 highland goats with known pedigrees and developed a new 3-step procedure for low-density SNP panels to support high-precision paternity testing. The RiceSNP50 array was used to genotype 195 rice inbred lines. A neighbor-joining (NJ) tree was constructed using the microarray typing results of these 195 rice inbred lines, with a accurate clustering into three populations (*indica*, *japonica*, and intermediate accessions) [25]. These studies have shown the effectiveness of SNP chips in population evolutionary analysis, paternity identification, and phylogenetic tree construction.

However, most SNP chips are biased towards use in breeding, with very few used exclusively for provenance identification. Given the current situation of antler deer breeding in China, there is an urgent need for an accurate and rapid method for the identification of pure sika deer, which can be applied during the preservation process. In this study, the first low-density genotyping chip for the identification of pure-bred sika deer was developed; this SNP chip can quickly and accurately distinguish sika deer from hybrid progeny and facilitate the protection of the germplasm resources of sika deer. This study provides a scientific basis for preventing the degradation of germplasm resources due to the hybridization of sika deer resources in China.

## Results

**Fig. 1:**
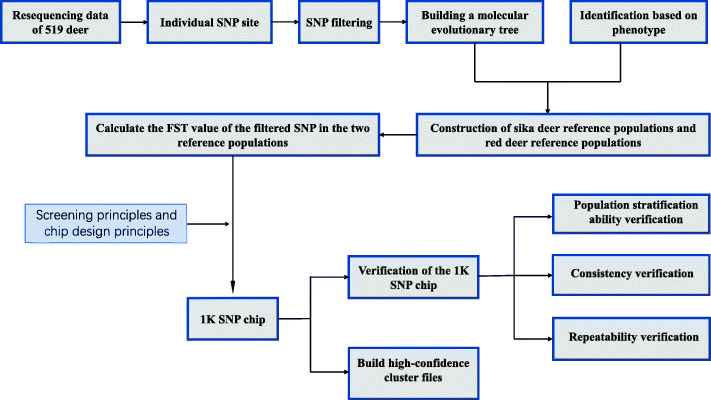
The roadmap for the design of the 1 K sika deer SNP array

The roadmap of development and validation of 1 K SNP chip is shown in Fig. 1, and the establishment of the 1 K SNP chip is indicated in the following paragraphs.

### Whole genome sequencing analysis

Sequencing of samples from all individuals yielded a total of 14.03 Tb of clean data with an average of 27.73 Gb per sample. Using the chromosome-level sika deer genome as the reference sequence, the clean reads obtained from the sequencing of each sample were aligned back to the genome, and average mapping rate, coverage, and sequencing depth of each sample were determined (Table 1).

**Table 1:** Sequencing quality

Projects	Clean reads	Average mapping rate	Sequencing depth (X)	Coverage
Groups				
Sika Deer	579,589,527	98.46%	26.78	98.68%
Red Deer	592,968,702	98.11%	26.26	99.46%
F1	208,431,775	99.19%	9.95	98.42%
F2	203,849,782	99.19%	9.81	98.43%
F3	206,270,153	98.85%	9.78	98.37%

### SNP screening and chip design

**Fig. 2:**
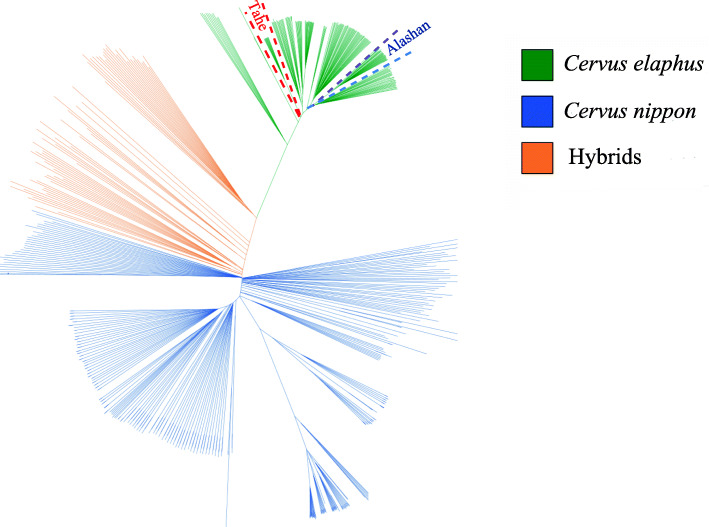
Phylogenetic tree of 519 samples. According to the linear sequence of the filtered SNP sites of the 519 resequencing samples, the conserved region sequences in all samples were screened, and the nearest-neighbor algorithm was used to construct a phylogenetic tree

**Fig. 3:**
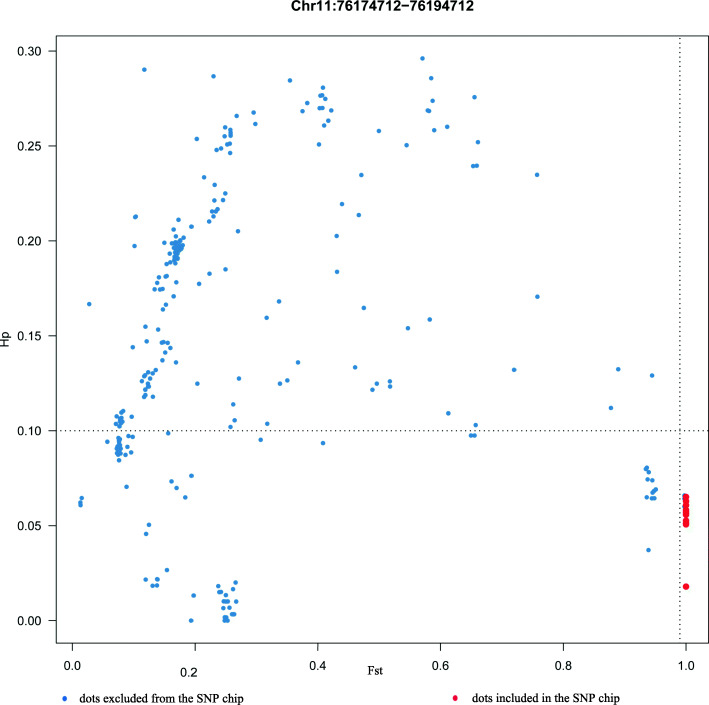
Fst values and heterozygosity of some SNPs (red dot) included in the 1 K SNP chip. The red dot indicates a SNP included in the SNP chip, and the blue dot indicates a SNP excluded from the SNP chip

The sequencing data were compared to the reference genome, and a total of 130,306,923 SNPs were detected. After hard filtering (see methods), 31,140,900 sites were selected for tree building (Fig. 2). The results showed that sika deer and red deer clustered separately at the two ends of the evolutionary tree. F1, F2, and F3 clustered between sika deer and red deer. According to the positions of individuals in the evolutionary tree, although three individuals (DF-81, LW-DD-057, and LW-CLW-40) showed phenotypes that matched those of sika deer, they clustered with hybrid deer, so they should be excluded from the sika deer population. Based on the molecular level and phenotypes results, 247 pure sika deer and 206 red deer were selected as the pure sika deer reference population and red deer reference population, respectively. The Fst values of all SNP loci in both reference populations and the heterozygosity of each locus were determined. There were 958,889 loci with Fst values greater than 0.95. According to the screening principles (see methods), 1000 SNP loci were finally selected. Figure 3 shows that some SNP sites (red dots) included in the SNP chip had high Fst values and low heterozygosity. The rest of chromosomes are shown in Additional file 1: Fig. S1. The average Fst of the 1000 SNP loci was 0.997, the minor allele frequency (MAF) was between 0.3277 and 0.3621 (with an average of 0.3483), and the average chip score was 0.99 (Additional file 2: Table S1). The annotation information of all SNP loci is provided in Table 2. A list of related genes of all SNPs that fall in the gene region (exon region and intron region) is given in the attachment (Additional file 3: Table S2).

**Table 2:** Annotation information of the 1 K SNP chip loci

Type of variation	Quantity	Ratio (%)
Upstream	26	2.6
Intronic	484	48.4
Intergenic	442	44.2
Exonic	25	2.5
Downstream	23	2.3

**Fig. 4:**
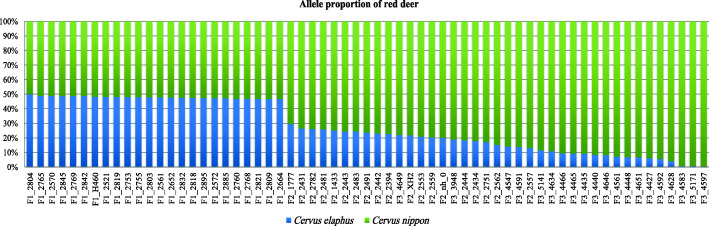
The average proportion of red deer alleles in hybrid samples. The two colors represent sika deer and red deer

According to Fig. 4, the average proportion of red deer alleles in the F1-generation samples was 0.48 (± 0.008), that in the F2-generation samples was 0.24 (± 0.02), and that in the F3-generation samples was 0.11 (± 0.05) (Additional file 4: Table S3). The gene content of red deer gradually decreased with the hybrid generation, generally reflecting the laws of statistical genetics.

### Improvement of genotyping chip accuracy

**Fig. 5:**
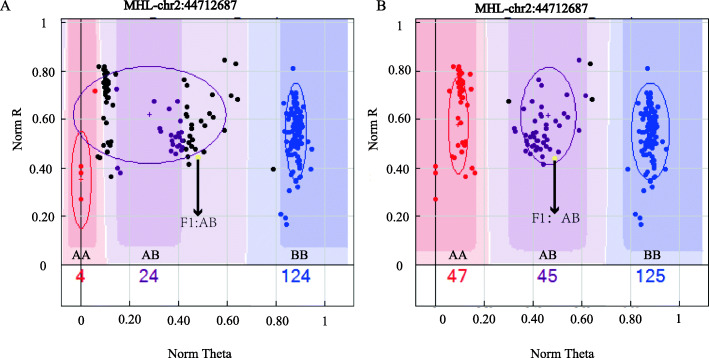
Corrected SNPs, where **A** and **B** indicate default clustering using GenomeStudio software and adjusted clustering, respectively

GenomeStudio software was used to perform cluster analysis on the genotyping signals detected by oligomer probes, resulting in three groups. In the first group, the default parameters could be used to clearly distinguish the genotypes of most samples (Additional file 5: Fig. S2). The second group consisted of markers for which some or all samples had uncalled genotypes. In addition, data for 4 SNPs were missing from all samples because these SNPs showed complex cluster graphs that could not be accurately clustered even with manual adjustment or a NormR > 0.2 (Additional file 6: Fig. S3). In the third group, some sites required adjustment to obtain accurate genotyping. Figure 5A is a clustering diagram automatically generated using only GenomeStudio software. F1 samples of a known genotype (AB) were not clustered to the corresponding position. To solve this problem, we resequenced samples with known genotypes to correct the genotyping results of the SNP chip and constructed high-quality clustering files. Through this adjustment, the F1 samples were correctly clustered to the corresponding positions, as shown in Fig. 5B.

### Verification of the 1 K array

**Fig. 6:**
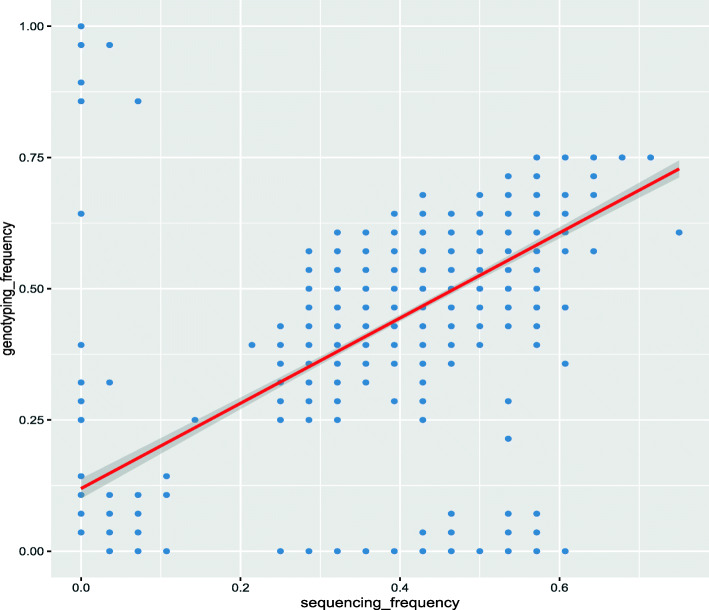
Evaluation of the accuracy of chip test results. Correlation between sequence-derived and genotype-derived allele frequencies. The scatter plot was created using the frequencies of sika deer 1 K genotypes derived from WGS

A significant correlation between the genotyping obtained by resequencing and the genotyping of the SNP chip at all loci was detected (r = 0.6507, *p* < 0.0001), as shown in Fig. 6. The average agreement was 93.48% (Additional file 7: Table S4). The genotyping results obtained for the same sample with different chips were consistent. Analysis of the SNP chip test data of 266 samples demonstrated that 973 sites were polymorphic. The 833 SNP sites remaining after filtering (see methods) were used for subsequent analysis. (Additional file 8: Fig. S4) The average MAF of the remaining loci was 0.38, the average detection rate of SNP loci was 98.7%, and the population average detection rate was 92% (F1)-95.30% (sika deer). These findings indicate that the genotyping results of the SNP chip are reliable.

**Fig. 7:**
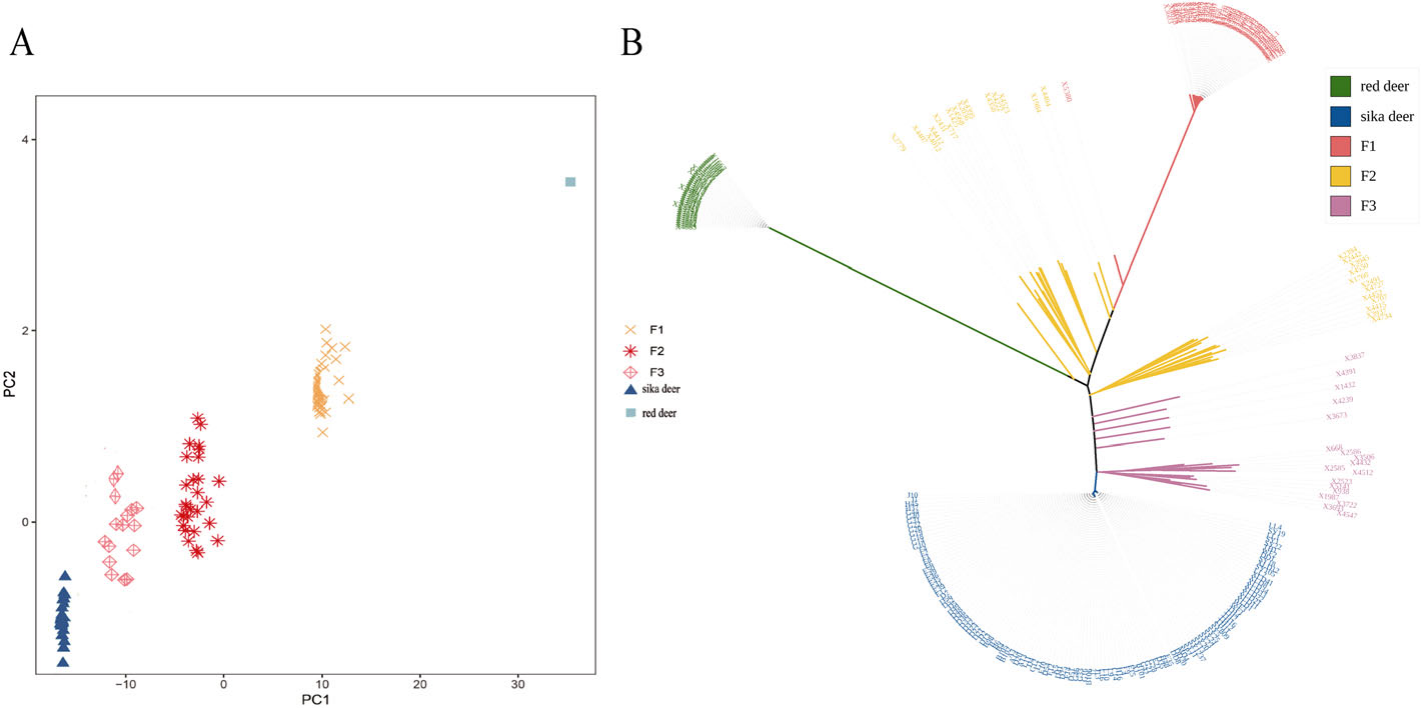
PCA and phylogenetic tree analysis of 266 test samples. Phylogenetic analysis of 266 samples based on the sika deer 1 K genotyping array. **A**: The PCA results of 5 groups. Each dot represents an individual, and different colors represent different groups. **B**: A neighbor-joining tree constructed using 833 polymorphic SNP markers

The genotyping data of these samples were analyzed by principal component analysis (PCA) (Fig. 7A). In the figure, the left side of the PC1 axis corresponds to sika deer, and the right side corresponds to red deer. The hybrid deer are located between the two deer species, and there is clear distinction among F1, F2, and F3. The results of the phylogenetic tree analysis (Fig. 7B) and the PCA were generally consistent. The cross-validation program of ADMIXTURE software can help select the best K value and perform cross-validation under the default setting (−-cv). The cross-validation error is lowest when K = 7 (Additional file 9: Fig. S5 A). The ADMIXTURE result (Additional file 9: Fig. S5 B) shows that when the ancestral components come from two populations of sika deer and red deer (K = 2), there are obvious differences between sika deer (red), red deer (blue), and hybrid deer, and the hybrids showed the same ancestry. When K = 3, the F1 hybrid deer is separated from the hybrid population and can be clearly distinguished from other hybrid offspring, while the F2 and F3 hybrid deer have a certain degree of mixing.

**Fig. 8:**
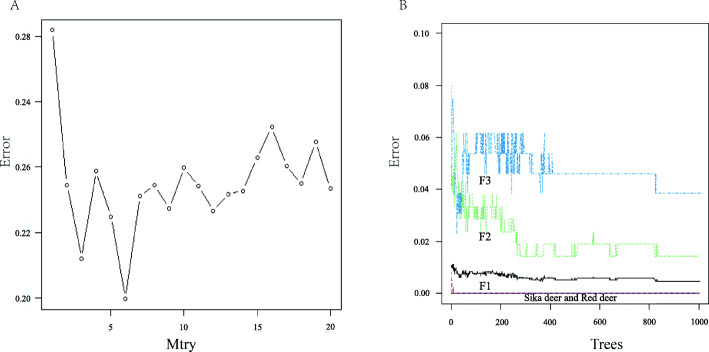
The relationship between random forest parameters and error rate

According to Fig. 8A, the error rate was the lowest when *Mtry* = 6. Thus, the number of preselected variables for each tree node was set to 6, and *Mtry* = 6 was selected to construct the random forest model. As shown in Fig. 8B, when *Mtry* = 6 and the number of decision trees was less than 400, the error of the model fluctuated greatly. When the number of decision trees was greater than 400, the model gradually stabilized, but there were still some fluctuations. Because the error rate of the model was lowest when the number of decision trees was 850, 850 was selected as the number of decision trees in the random forest. Then, the trained random forest model was used for classification, and the out-of-bag (OOB) error rate of these loci was 4.76%, indicating that the accuracy of assigning an unknown individual to its corresponding population was 95.24%. In the receiver operating characteristic (ROC) graph, the area under the curve (AUC) was 0.941, indicating that the model had a better classification effect.

## Discussion

The sika deer subspecies currently found in China include *Cervus nippon hortulorum*, *Cervus nippon sichuanicus*, *Cervus nippon kopschi*, and *Cervus nippon taiouanus* [26]. After a long period of domestication, *Cervus nippon hortulorum* has formed a domestic sika deer population, including 7 breeds (Shuangyang sika deer, Dongda sika deer, Aodong sika deer, Dongfeng sika deer, Xifeng sika deer, Xingkai Lake sika deer, and Siping sika deer) and a Changbai Mountain strain. Among these breeds (strains) of sika deer, Shuangyang sika deer have the characteristics of high yield, stable genetic performance, strong adaptability, medium size, no obvious backline and throat spots, short and thick eyebrows and red hair; Siping sika deer exhibit a short and thick antler trunk and a mostly ingot-type mouth with red-yellow antlers; Dongfeng sika deer are characterized by strong limbs with sparse and large motifs, a thick antler body, and a notably round mouth; Dongda sika deer have a strong, thick body, long branch antler trunk, and short and large motifs. The common characteristics of these varieties (strains) are high production performance and stable genetic performance. These varieties have been widely used to improve low- and medium-yield deer herds, and are currently the most commonly used populations for breeding and cross-breeding [27]. *Cervus nippon sichuanicus*, *Cervus nippon kopschi*, and *Cervus nippon taiouanus* are primarily distributed in the wild environment, their degree of domestication is low, and they are rarely used in cross-breeding [28]. At present, the most common crossbreeding method involves using *Cervus nippon hortulorum* as the female parent and *Cervus canadensis songaricus*, *Cervus elaphus xanthopygus*, or *Cervus elaphus yarkandensis* as the male parent [29].

The phylogenetic tree was constructed by using the genetic distances between individuals belonging to populations analysed. This method is often used for genetic diversity analysis and parental line selection [25]. The phylogenetic trees of the five populations are shown in Fig. 2. The hybrid deer population clustered between the sika deer and red deer, and different species/subspecies of sika deer and red deer clustered together according to geographical location, such as red deer in Tahe and Alashan. Japanese sika deer showed similar results: the sika deer populations in northern and southern Japan were located on different branches and later formed a large branch, which further supports the view that the Japanese population is derived from at least two pedigrees [30]. In this study, phenotypes and molecular evolutionary trees were jointly considered, and 247 purebred sika deer and 206 red deer were selected as the sika deer reference population and red deer reference population. The SNP loci were strictly screened according to their Fst values by using a customized algorithm, which ultimately yielded a total of 1000 SNP sites for chip development.

Figure 4 shows that as the generation of crosses progresses, the offspring of the hybrids contain a decreasing number of alleles specific to red deer and an increasing number of alleles specific to sika deer. This phenomenon is observed because the current hybrid deer are mostly produced by progressive crosses between sika deer and red deer. The alleles of the hybrid offspring specific to red deer did not decrease by exactly 50, 25, and 12.5%, which may be due to the difference in chromosome type between the red deer and sika deer [31].. Ba et al., [32] employed double-digest restriction-site associated DNA sequencing (ddRAD-seq) technology and detected 320,000 genome-wide SNPs in 30 captive individuals (7 sika deer, 6 red deer and 17 F1 hybrids), screening out 2015 potential diagnostic SNP markers that can be used to evaluate or monitor the degree of hybridization between sika deer and red deer. However, the experimental population in the study was small, and no large group (30 individuals in only three populations) verification was carried out. Compared to the research of Ba and collaborators [32], this study employed whole-genome sequencing, and the sequencing depth and coverage were considerably higher than those of ddRAD-seq. Moreover, the size of the reference population selected for this study was relatively large (250 sika deer, 206 red deer, 23 F1, 20 F2, and 20 F3), and the accuracy of the sites was verified using 266 verification samples (5 populations). Therefore, the accuracy of the results of this study is greater than that of the previous study.

To verify the ability of the 1 K SNP chip to detect population structure, a total of 266 samples of sika deer, red deer, and hybrid deer were tested, and the average detection rates of the populations were 92–95.30%. In all individuals, 97.89% of the SNP loci were polymorphic, which indicates that the 1 K sika deer SNP chip can be used to determine the genetic variation among sika deer, red deer, and hybrid deer. According to the PCA results, sika deer, red deer, and hybrid deer were clustered into different positions, and the hybrid deer were arranged from left to right according to the number of consanguinity relatives that were sika deer. The results of the random forest model showed that the accuracy of the 1 K sika deer SNP chip in identifying unknown individuals was 95.24%. Therefore, the 1 K sika deer SNP chip can accurately identify the provenance of the sample to be tested.

There are currently few SNP chips available for deer. Bixley et al., [33] used reduced representational sequence technology to screen 768 SNPs for the development of a Golden Gate (Illumina™) SNP chip. The author assembled a mapping pedigree to implement quality control of these and other SNPs and to produce a genetic map. This SNP chip will be a new parentage assignment and breed composition panel. Rowe et al., [34] developed an Illumina SNP chip for New Zealand deer breeding. The chip contains 132 SNP markers for paternity testing. These markers can identify the New Zealand deer breeds. For deer, 1000 randomly selected SNPs were used to successfully assign samples to genetic groups based on their main genetic and geographic differences. Brauning et al., [35] used next-generation sequencing to sequence seven *Cervus elaphus* (European red deer and Canadian elk) individuals and align the sequences to the bovine reference genome build UMD 3.0. The authors identified 1.8 million SNPs meeting the Illumina SNP chip technical threshold. Genotyping of 270 SNPs on a Sequenom MS system showed that 88% of the identified SNPs could be amplified. Compared with the abovementioned SNP chips, the 1 K sika deer SNP chip is mainly used to identify domestic deer in China. In addition, in the past, the reference genome of bovines was used for alignment. For the first time, in this research, the sika deer genome was used for alignment to ensure the accuracy of microarray typing results.

## Conclusion

In this study, morphological identification combined with molecular-level analysis was used to establish a reference population. A total of 247 purebred sika deer and 206 red deer were selected as sika deer reference population and red deer reference population. The Fst value of each SNP site in those two reference populations was calculated. The screening and customization algorithm yielded 1000 SNP sites for the development of the microarray, and the distribution of these 1000 sites in the hybrid deer was examined, producing a result in line with the laws of statistical genetics. In terms of 1 K SNP chip verification, the consistency between the microarray genotyping results and the high-throughput sequencing results was 93.48%, and the consistency of the sequencing results between different chips and for the same individual on the same chip was 100%, indicating that the microarray genotyping results were reliable. In addition, machine learning algorithms (random forest) and PCA were used to verify the population stratification ability of the SNP sites on the 1 K SNP chip. The accuracy of the 1 K sika deer SNP chip in identifying unknown individuals was as high as 95.24%. In summary, the 1 K sika deer SNP chip can accurately identify pure sika deer, hybrid deer, and red deer, providing technical support for the identification of pure sika deer provenance and laying a solid foundation for the subsequent breeding of sika deer.

## Methods

### Ethics statement

All procedures concerning animals were organized in accordance with the guidelines of care and use of experimental animals established by the Ministry of Agriculture of China, and all protocols were approved by the Institutional Animal Care and Use Committee of Institute of Special Animal and Plant Sciences, Chinese Academy of Agricultural Sciences, Changchun, China.

### Animals

To increase the accuracy of identification, four existing Chinese sika deer subspecies, Russian sika deer, Japanese sika deer, and all existing Chinese red deer subspecies and North American subspecies were selected. Specifically, the red deer were from Xinjiang, Northeast China, Gansu, Qinghai, Sichuan and Tibet, and the sika deer were from Northeast China, South China, Sichuan, Taiwan, Russia and Japan. See Table 3 for detailed sample information. The appearance of different groups is shown in Additional file 10: Fig. S6 (sika deer and red deer) and Additional file 11: Fig. S7 (F3-generation). Finally, a total of 519 sample (250 sika deer, 206 red deer, 23 F1 hybrids, 20 F2 hybrids, and 20 F3 hybrids) were randomly selected, and phenotypic identification (head length, coat color, backline, tail spots, throat spots and hip spots) was performed following [36].

**Table 3:** Resequencing sample information

Species	Subspecies/species	Quantity
Red Deer	*Cervus canadensis asiaticus*	35
Red Deer	*Cervus elaphus alashanicus*	28
Red Deer	*Cervus canadensis*	9
Red Deer	*Cervus elaphus macneilli*	10
Red Deer	*Cervus elaphus xanthopygus*	30
Red Deer	*Cervus elaphus kansuensis*	20
Red Deer	*Cervus elaphus*	16
Red Deer	*Cervus elaphus yarkandensis*	11
Red Deer	*Cervus canadensis songaricus*	11
Red Deer	*Cervus elaphus wallichii*	20
Red Deer	*Cervus elaphus xanthopygus*	16
Sika Deer	Dongda Sika Deer	16
Sika Deer	Aodong Sika Deer	5
Sika Deer	Dongfeng Sika Deer	15
Sika Deer	Russian Wild	16
Sika Deer	Russian Domesticated	6
Sika Deer	*Cervus nippon kopschi*	8
Sika Deer	*Cervus nippon yesoensis*	11
Sika Deer	*Cervus nippon aplodontus*	11
Sika Deer	*Cervus nippon pulchellus*	14
Sika Deer	*Cervus nippon yakushimae*	9
Sika Deer	*Cervus nippon*	3
Sika Deer	Shuangyang Sika Deer	11
Sika Deer	*Cervus nippon sichuanicus*	8
Sika Deer	Siping Sika Deer	13
Sika Deer	*Cervus nippon taiouanus*	2
Sika Deer	Tonghua Sika Deer	17
Sika Deer	Xingkai lake Sika Deer	10
Sika Deer	*Cervus nippon dybowskii*	74
Sika Deer	Dunhua Sika Deer	1
Hybrid Deer	First-generation Hybrid Deer	23
Hybrid Deer	Second-generation Hybrid Deer	20
Hybrid Deer	Third-generation Hybrid Deer	20

In addition, there are 266 deer used for 1 K SNP chip verification (125 sika deer, 39 red deer, 56 F1, 29 F2, 17 F3). These deer are provided by Jilin Keda Co., Ltd.

A total of 785 samples were raised in captivity, all of which were derived from wild-caught deer and were maintained under closed flock breeding for 5–50 generations. Chemical anesthesia was used during deer catching. Lumianning injection (070011777, Jilin Huamu Animal Health Products Co., Ltd., China), an anesthetic, was administered intramuscularly at 1 ml per 100 kg of body weight, and peripheral vein blood of each sample was collected fresh and stored at − 20 °C until DNA extraction.

### Main instruments and reagents

The centrifuge (Sigma 1-14 K) was purchased from Sigma-Aldrich (Shanghai) Trading Co., Ltd.;The electrophoresis instrument (EPS-300) was purchased from Shanghai Tianneng Technology Co., Ltd., and the gel imaging system (SYSTEMGelDocXR+IMAGELA) was purchased from Bio-Rad Life Medical Products (Shanghai) Co., Ltd.

The blood genomic DNA extraction kit (DP348–03) was purchased from Tiangen Biochemical Technology (Beijing) Co., Ltd.; Isopropanol, absolute ethanol, agarose, 50× TAE, 6× loading buffer, and DNAMarker (e.g., DL15000) were purchased from Shanghai Biological Engineering Co., Ltd.

### Whole-genome resequencing (database construction)

Blood was collected from the jugular vein of the experimental animals, and a blood genomic DNA extraction kit (DP348–03) and a high-throughput magnetic bead extraction system were used to extract the genomic DNA from the blood samples. The DNA obtained was subjected to Illumina HiSeq 2000 sequencing (Beijing Nuohe Zhiyuan Biological Information Technology Co., Ltd.).

### Discovery and screening of specific sites

Previous studies have pointed out that the morphological characteristics of deer may not correctly reflect their evolutionary relationships, and the phylogenetic relationship between deer species and subspecies should be analyzed by combining the results of morphological studies at the molecular level [37]. Therefore, to screen out specific SNP sites, the reference population of this study was established on the basis of phenotypic and molecular identification. Identification at the molecular level was performed using NGS QC Toolkit (default parameters) [38] to filter the genotyping data of resequenced samples in order to remove reads meeting the following three conditions: 1. Reads containing linker sequences, 2. Single-end reads of N for which the number of bases exceeded 10% of the total number of read bases, and 3. Single-end reads with low-quality (quality value less than 5) bases that exceeded 50% of the length of the read. BWA-MEM (v0.7.12) [39] software was used to compare the filtered reads to the sika deer reference genome (mhl_v1.0), and SAMtools (v1.9) software [40] was used to sort bam files to remove duplicates. Next, GATK4.0.2.1 software was utilized for mutation detection [41], and the filtering conditions (−filter “QD < 2.0” –filter-name “QD2”, −filter “QUAL < 30.0” –filter-name “QUAL30”, −filter “SOR > 3.0” –filter-name “SOR3”, −filter “FS > 60.0” –filter-name “FS60”, −filter “MQ < 40.0” –filter-name “MQ40”, −filter “MQRankSum < -12.5” –filter-name “MQRankSum-12.5”, −filter “ReadPosRankSum < -8.0” –filter-name “ReadPosRankSum-8”) were applied to perform hard filtering. Meanwhile, VCFtools-0.1.13 [42] was used to eliminate sites; detect SNPs with a missing rate greater than 0.1, locus coverage less than 5X, and locus quality less than 30; and perform less hard filtering. According to the linear sequence of filtered SNP sites, Gblocks 0.91 software [43] was employed to screen the conserved region sequences in all samples, and TreeBeST 1.9.2 [44] software was used to construct a phylogenetic tree with the nearest-neighbor algorithm.

At the same time, phenotypic identification of individuals was performed according to the body appearance of all samples (head length, coat color, backline, tail spots, throat spots and hip spots), and the sika deer reference population and red deer reference population were finally selected based on the cluster position and phenotypic of the samples.

The Fst between populations is a measure of population differentiation and genetic distance with a value between 0 and 1. The greater the differentiation index is, the greater the difference is [45]. To screen the specific sites of red deer, the Fst value of each SNP site between the red deer reference population and the sika deer reference population was calculated by VCFtools-0.1.13 [42], and only sites with an Fst > 0.95 were retained. At the same time, it was required that the selected SNP loci be mutually exclusive in the genotypes of red deer and sika deer. In other words, the frequency of genotype AA in red deer was 1, and the frequency of CC in sika deer was 1, with the highest priority. We further filtered the candidate SNP sites according to the customization requirements of the microarray. The filter conditions include the following: 1. The flanking sequence of the site (within 50 bp) had no interference SNP, and 2. All [G/C] or [A/T] conversion sites were deleted; that is, only SNP sites of the transversion type were retained.

To observe the genetic stability of the selected SNP loci, we used the sequenced F1, F2, and F3 generation samples as the test samples. Based on the 1000 selected loci, we calculated the frequency of the specific loci in the hybrid deer and calculated the proportion of red deer genetic content in each hybrid sample. (Additional file 12: Table S5).

### Designing the 1 K genotyping array

Illumina chips have two types of SNP sites [46]: single-bead type II SNPs (A/C, A/G, T/C, and T/G) and two-bead SNPs. For type I SNPs (A/T, C/G), we selected only type II SNPs to maximize the number of genotyping polymorphisms. The selected candidate SNPs (4 K) of four times the target size were provided to the Illumina company to design a 51-mer sense nucleotide sequence. The target SNP site was located at the 26th position. A customized algorithm was used to calculate each submitted SNP sequence. SNPs with scores less than 0.6 were removed [47]. To ensure the accuracy of the results, each SNP was tested with three probes. During the analysis, the signals from the three detections were summarized, and a single SNP was provided for each SNP signal estimation.

### SNP marker analysis and cluster file construction

Illumina synthesized 995 markers and used GenomeStudio software (v2011.1, Illumina, Ink) to perform cluster analysis on the genotyping data of the SNP chip results for the test sample. At the same time, to increase the accuracy of the results, 1 K SNP chip genotyping was used for resequencing samples, and the clustering diagram of chip products was optimized and adjusted based on the high-confidence (e.g., library sequencing depth ≥ 10×) genotyping results of resequencing analysis [48]. The resequencing samples included 10 F1, 9 F2, and 9 F3 samples for a total of 28 samples. (Additional file 13: Table S6).

### Verification of the chip

First, to verify the accuracy of microarray genotyping, we selected 28 samples (Additional file 13) that had been resequenced in the previous stage and used microarrays for genotyping to assess the consistency of the two results for each individual and the correlation of all sites. At the same time, four DNA samples from different individuals were selected and repeated three times on each chip and on different chips to determine the repeatability of the chip.

The second step was to investigate the ability of 1 K SNP chip to detect population structure. We chose 266 deer with a clear pedigree relationship (three generations) and no genetic relationships (see Table 4 for details). These verification samples were genotyped using 1 K SNP chip. For the genotyping data of the sample, ensure that the SNPs to be analyzed met Hardy-Weinberg equilibrium (HWE)(*P < 0.01*), we filtered the SNP sites according to a call rate > 95% and an MAF > 0.05 [22], and we subsequently deleted samples with a genotype deletion rate of more than 10% by SNP Variation Suite v7 (SVS; Golden Helix Inc., Bozeman, Montana: www.goldenhelix.com) [49]. According to the genotyping data of the sample, PCA was performed using the prcomp function in R-4.0.2 [50], and then the ggplot2 package was used for mapping [51]. TreeBeST software was used to construct the NJ tree [52], and 1000 bootstrap replicates were employed. Drawing was performed with iTOLv4 [53]. ADMIXTURE software was used to analyze the population structure based on the Bayesian model [54], and the clustering model was constructed based on the 1 K SNP chip genotyping data of 266 verification samples. This was performed by assuming the number of different ancestral sources K (1–8), inferring the ancestral composition of all samples in the population, determining the attribution of each individual, and studying the population structure of 266 verification samples.

**Table 4:** Chip verification sample information

population	Number	Collection location
Sika Deer	125	Jilin Jiutai
Red Deer	39	Jilin Jiutai
F1	56	Jilin Jiutai
F2	29	Jilin Jiutai
F3	17	Jilin Jiutai

To ensure the reliability of the SNP sites, we also used machine learning algorithms (random forest model) to evaluate their accuracy [55]. From each population, 30% of the samples were randomly selected to be used as the test set for the final classification effect test, and the remaining 70% were used as the training set. The random forest model has two important parameters: the number of decision trees (ntree) and the number of split node preselected variables (*Mtry*). Appropriate parameters are chosen according to the relationship between the parameters and the error rate. The “randomForest” package in R 4.0.2 software was used to construct a random forest model [56]. The SNP site date were applied for interval evaluation during the process of random forest generation and to obtain the corresponding OOB error rate [57]. An OOB error rate of 0 indicated that these sites could be used to accurately classify each sample.

## Supplementary Information

**Additional file 1 Fig. S1.** Fst values of SNP sites on different chromosomes.

**Additional file 2 Table S1.** MAF, Fst and score information of 1000 SNP sites.

**Additional file 3 Table S2.** Gene information of SNP chip locus (exon region and intron region).

**Additional file 4 Table S3.** The proportion of red deer genetic content in each hybrid sample.

**Additional file 5 Fig. S2.** A-F indicate the SNP sites that could be accurately classified by the default parameters of GenomeStudio (those with the first and second patterns (Fig. S2A, B). All formed a single cluster (AA, 00, 00**;** 00, 00, BB), representing a monomorphic locus. Those with the third and fourth patterns (Fig. S2C, D, E, F) were markers that showed three (AA, AB, AB) and two (AA, 00, BB**;** AA, AB, 00) clearly definable clusters.

**Additional file 6 Fig. S3.** A, B indicate the SNP sites that could not be accurately classified even with software adjustment.

**Additional file 7 Table S4.** A correlation between the genotyping obtained by resequencing and the genotyping of the SNP chip.

**Additional file 8 Fig. S4.** Venn diagram of sites for analysis. As can be seen from the figure, a total of 991 chip sites have been detected, 973 for MAF > 0.05, 850 for Call Rate > 95%, and 833 for SNP that meets MAF > 0.05 and Call Rate > 95%.

**Additional file 9 Fig. S5.** The results of population genetic structure analysis using ADMIXTURE software. A: Cross-validation error rate corresponding to different K values. B: Clustering results corresponding to different numbers of clusters (K value).

**Additional file 10 Fig. S6.** Photos showing the phenotypes of red deer and sika deer.

**Additional file 11 Fig. S7.** Photos showing the phenotypes of hybrid deer.

**Additional file 12 Table S5.** The frequency of the specific loci in the hybrid deer.

**Additional file 13 Table S6.** Sequencing quality information of samples used to correct 1 K SNP chip results.

## Data Availability

The original data is stored in the Special Resources Protection and Utilization Innovation Team of the Special Products Research Institute of the Chinese Academy of Agricultural Sciences (Changchun, Jilin). The datasets generated and analysed during the current study are not publicly available, Because the relevant data is under patent application. It will be disclosed when the patent application is available.

## Abbreviations

F1: First-generation Hybrid Deer
F2: Second-generation Hybrid Deer
F3: Third-generation Hybrid Deer
SNP: Single Nucleotide Polymorphism
PCA: Principal Component Analysis
RFLP: Restriction Fragment Length Polymorphism
RAPD: Random Amplified Polymorphic DNA
ALFP: Amplified Fragment Length Polymorphism
SSR: Simple Sequence Repeat
ISSR: Inter-simple Sequence Repeat
GWAS: Genome-Wide Association Study
MAF: Minor Allele Frequency
Fst: Fixation Index
