# Supplementary figures and images for: Development and validation of a 1 K sika deer (Cervus nippon) SNP Chip
Source: BMC Genom Data. 2021 Sep 17;22:35. doi: 10.1186/s12863-021-00994-z (PMC8447661; doi:10.1186/s12863-021-00994-z)

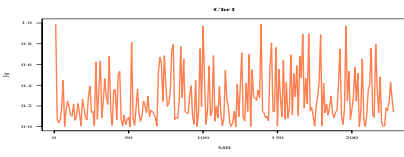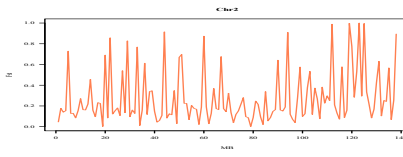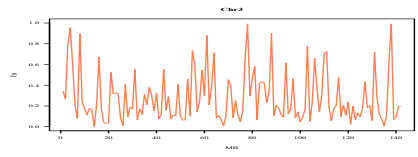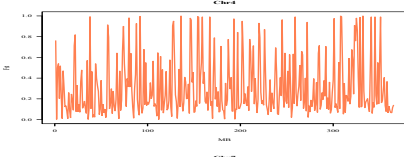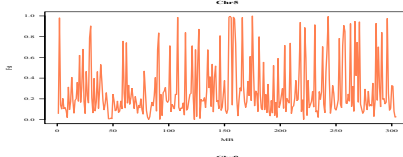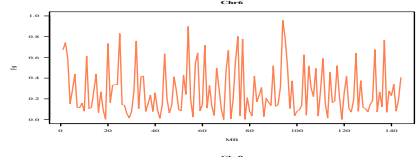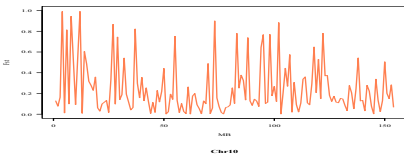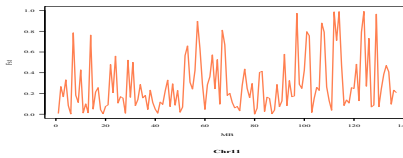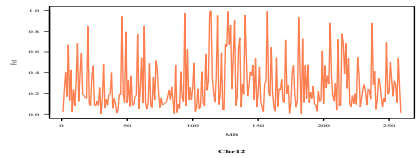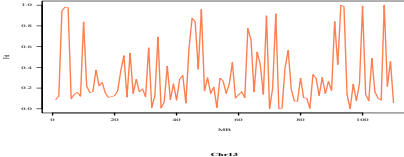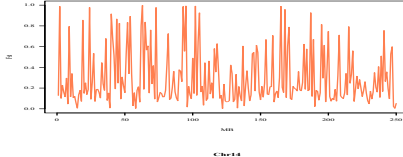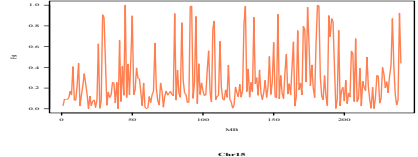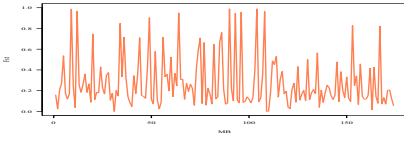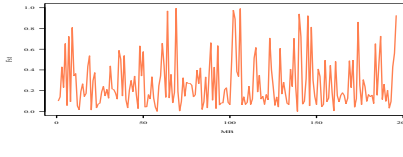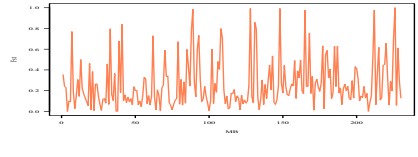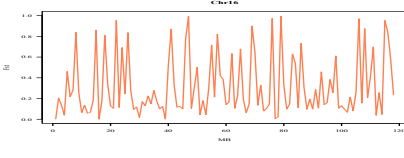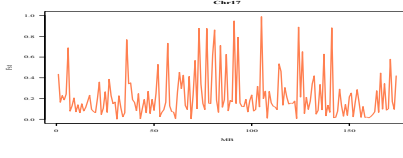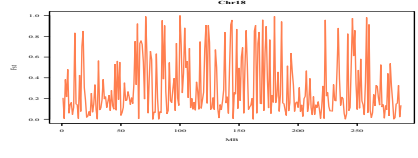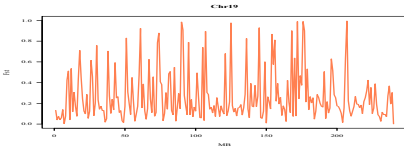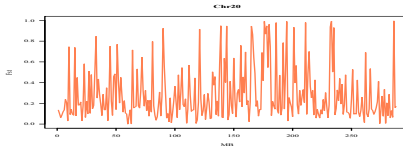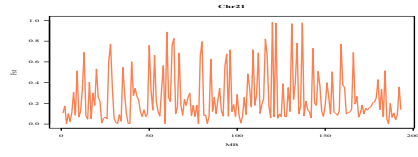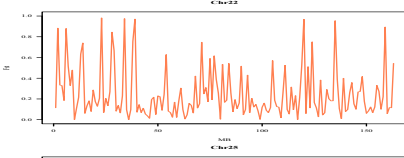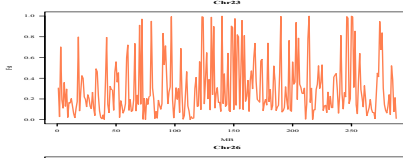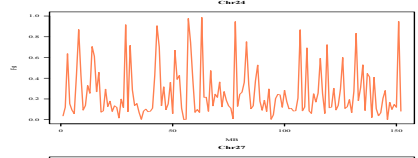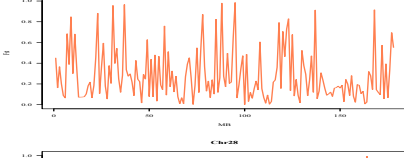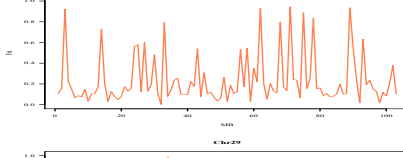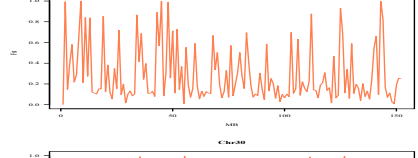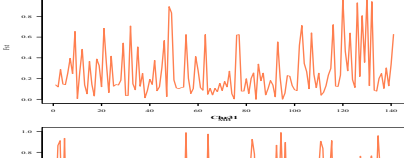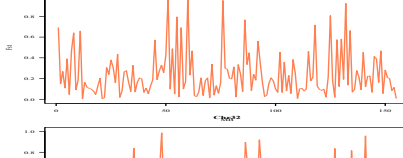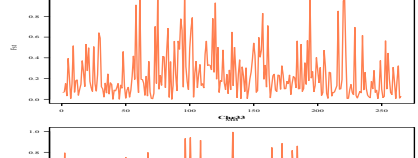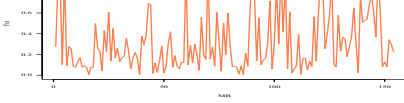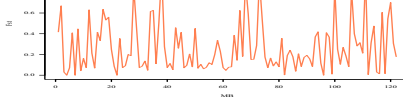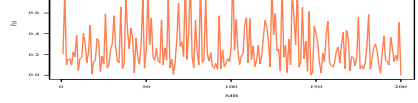

Supplement: Supplementary file 1 — Additional file 1 Fig. S1. Fst values of SNP sites on different chromosomes. [file 12863_2021_994_MOESM1_ESM.pdf]

a

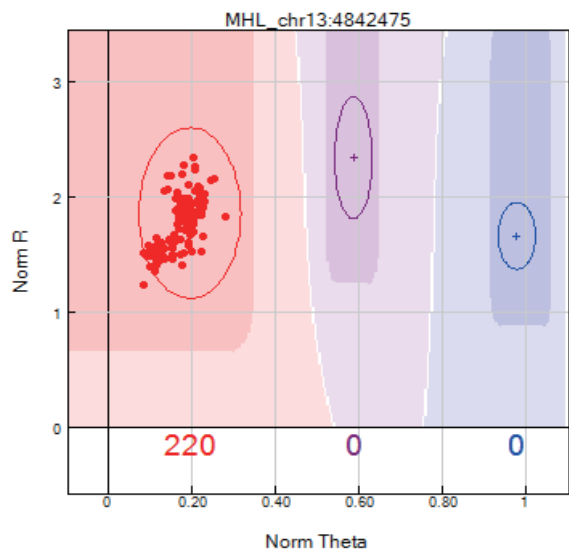

b

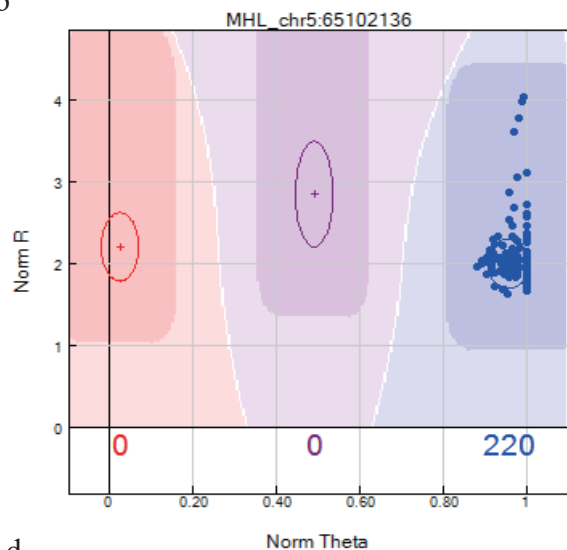

c

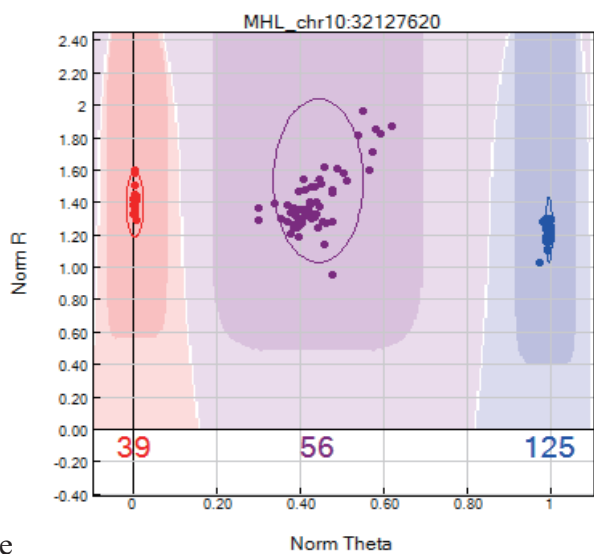

d

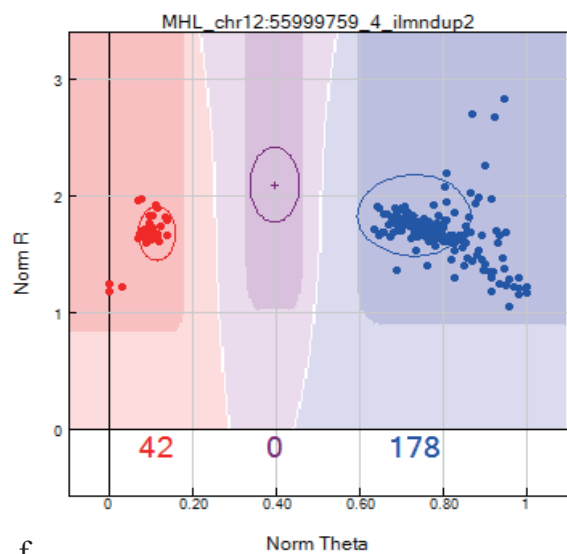

e

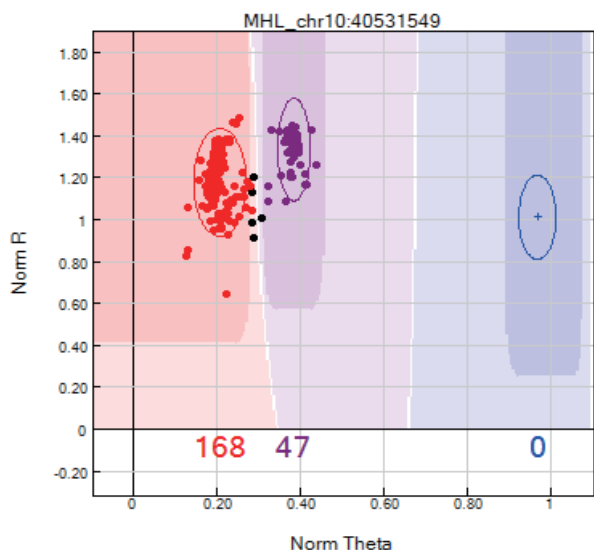

f

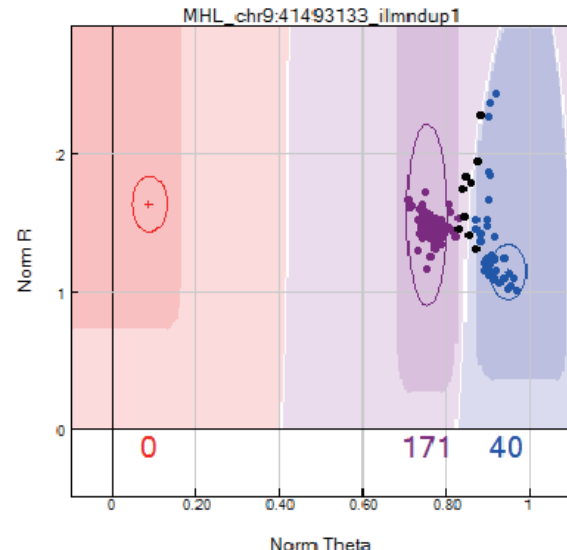

Supplement: Supplementary file 5 — Additional file 5 Fig. S2. A-F indicate the SNP sites that could be accurately classified by the default parameters of GenomeStudio (those with the first and second patterns (Fig. S2A, B). All formed a single cluster (AA, 00, 00; 00, 00, BB), representing a monomorphic locus. Those with the third and fourth patterns (Fig. S2C, D, E, F) were markers that showed three (AA, AB, AB) and two (AA, 00, BB; AA, AB, 00) clearly definable clusters. [file 12863_2021_994_MOESM5_ESM.pdf]

a

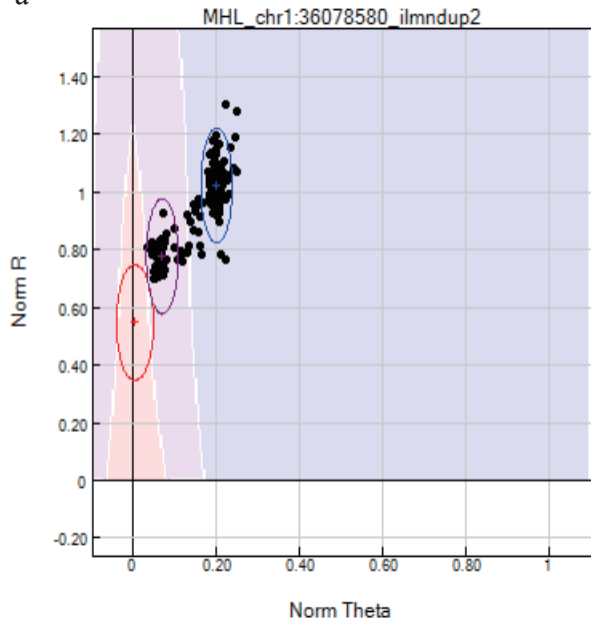

b

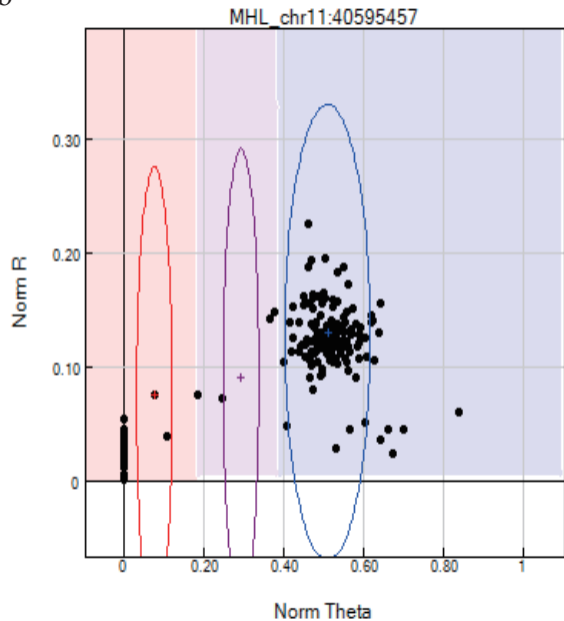

Supplement: Supplementary file 6 — Additional file 6 Fig. S3. A, B indicate the SNP sites that could not be accurately classified even with software adjustment. [file 12863_2021_994_MOESM6_ESM.pdf]

MAF>0.05

Detected

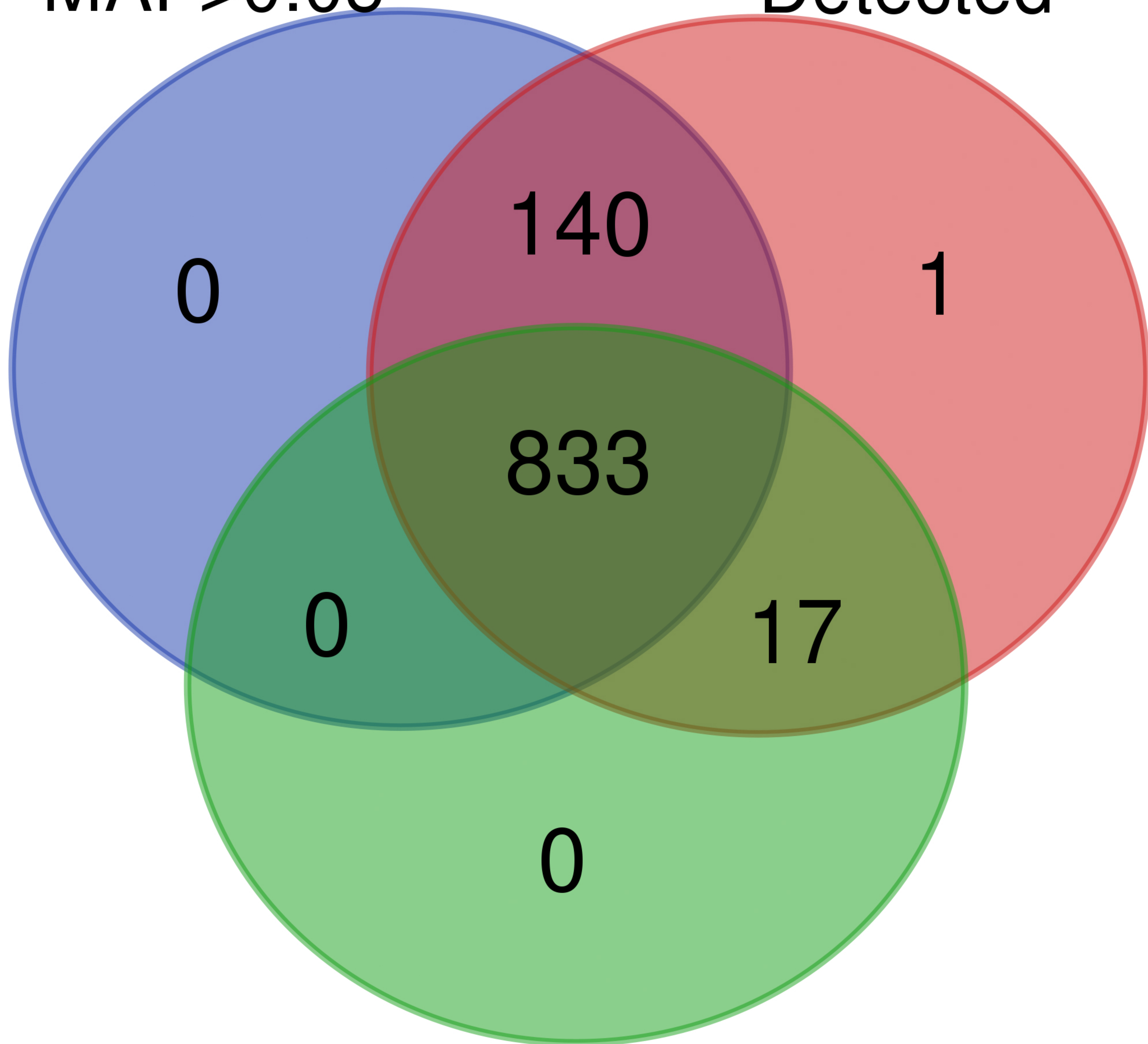

Call Rate>95%

Supplement: Supplementary file 8 — Additional file 8 Fig. S4. Venn diagram of sites for analysis. As can be seen from the figure, a total of 991 chip sites have been detected, 973 for MAF > 0.05, 850 for Call Rate > 95%, and 833 for SNP that meets MAF > 0.05 and Call Rate > 95%. [file 12863_2021_994_MOESM8_ESM.pdf]

A

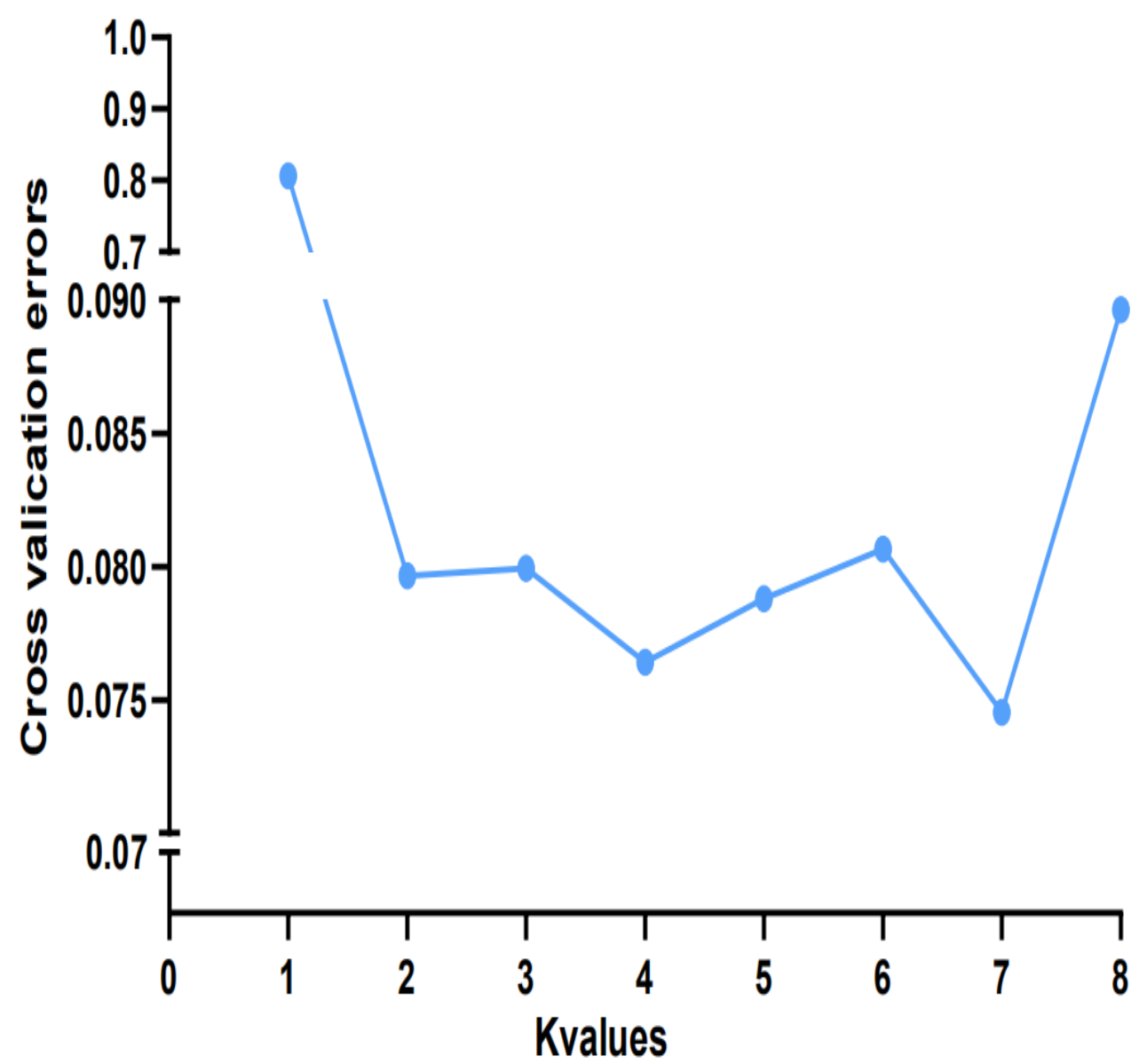

B

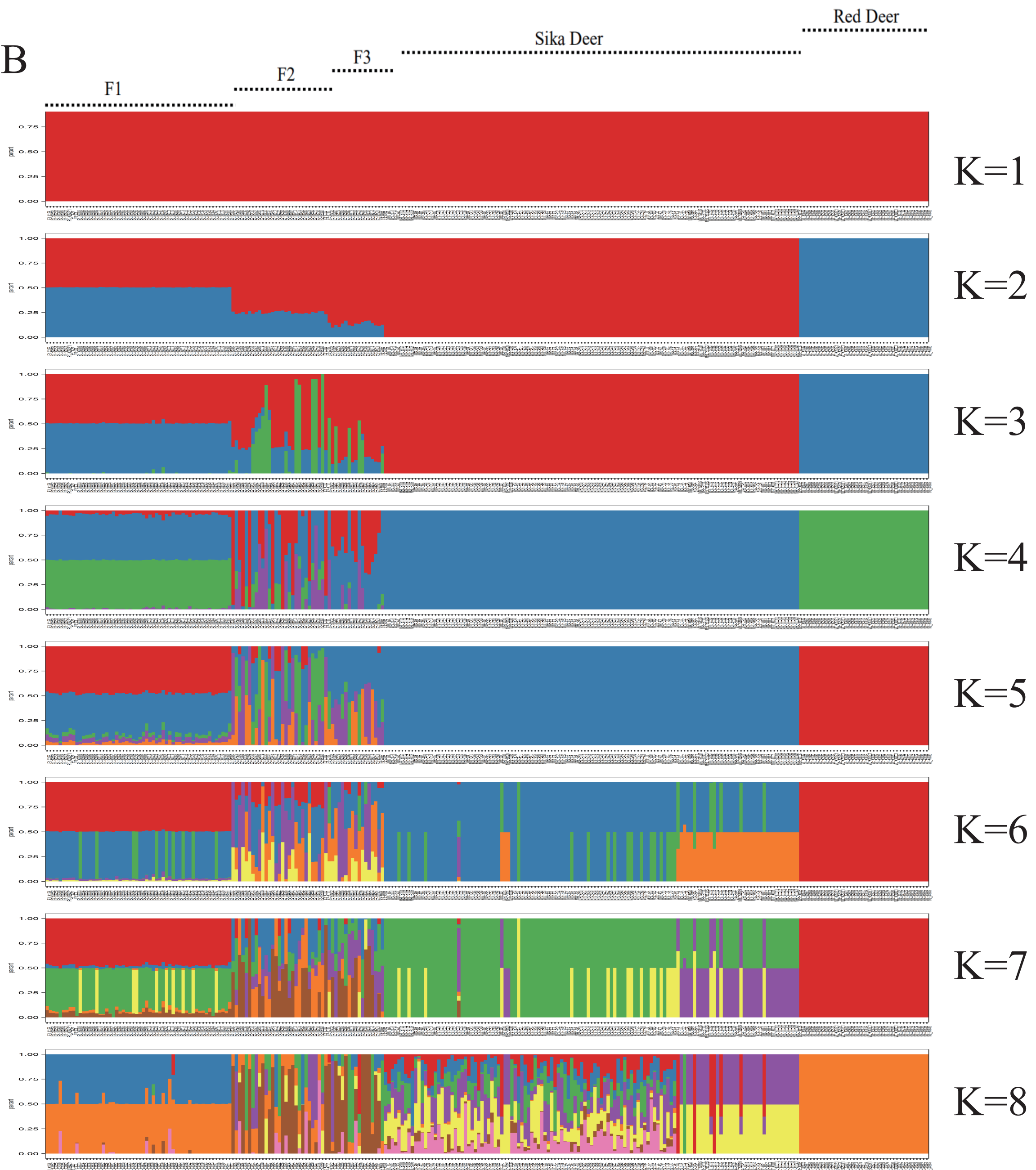

Supplement: Supplementary file 9 — Additional file 9 Fig. S5. The results of population genetic structure analysis using ADMIXTURE software. A: Cross-validation error rate corresponding to different K values. B: Clustering results corresponding to different numbers of clusters (K value). [file 12863_2021_994_MOESM9_ESM.pdf]

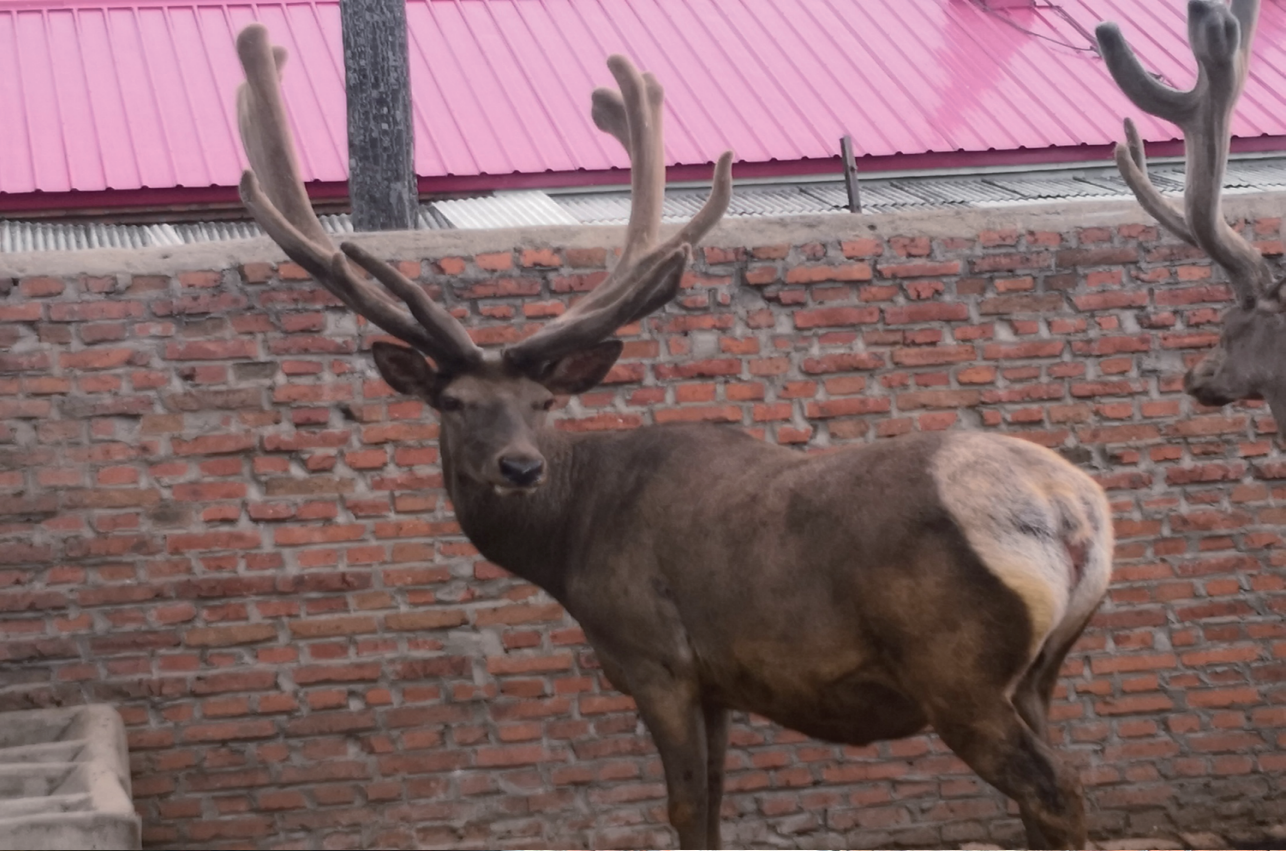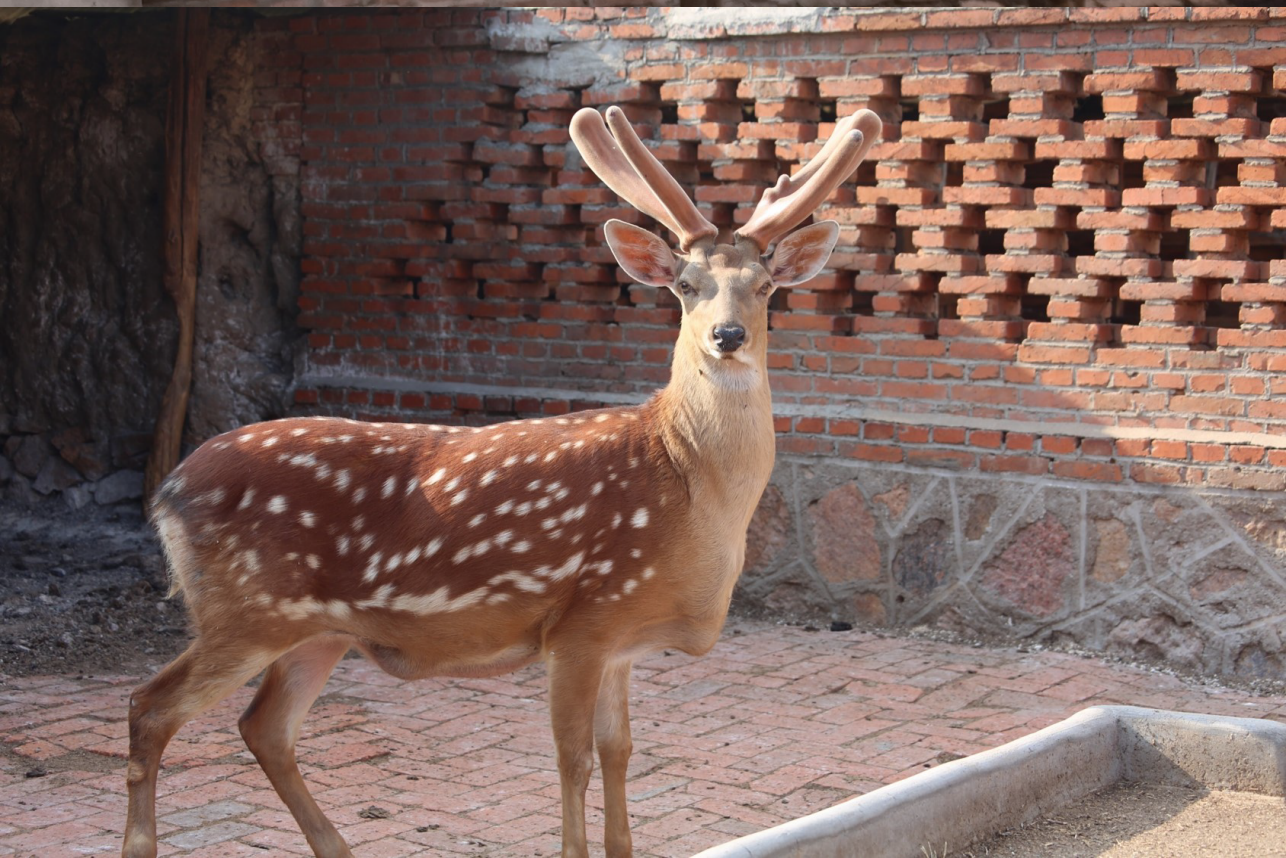

Supplement: Supplementary file 10 — Additional file 10 Fig. S6. Photos showing the phenotypes of red deer and sika deer. [file 12863_2021_994_MOESM10_ESM.pdf]

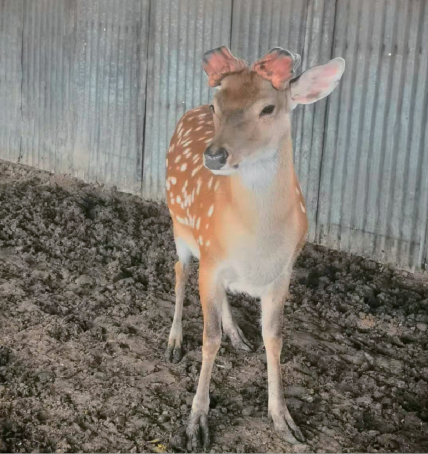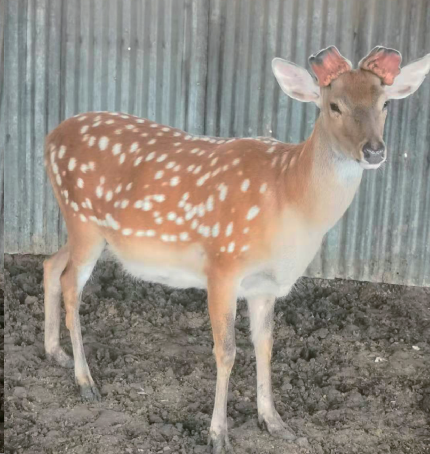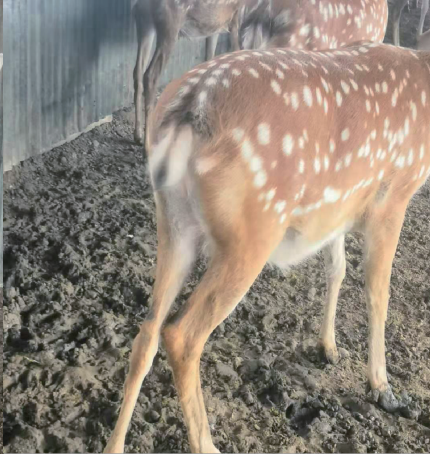

Supplement: Supplementary file 11 — Additional file 11 Fig. S7. Photos showing the phenotypes of hybrid deer. [file 12863_2021_994_MOESM11_ESM.pdf]
